# Construction and verification of a diagnostic nomogram for heart failure in hypertrophic cardiomyopathy based on conventional clinical indicators: a case-control study

**DOI:** 10.1186/s43044-026-00742-7

**Published:** 2026-05-08

**Authors:** Yu Li, Ziqi Duan, Jinlei Li, Bingxin Cheng, Fen Ai, Zhen Chen

**Affiliations:** 1 Department of Emergency Medicine, The Central Hospital of Wuhan, Tongji Medical College, Huazhong University of Science and Technology, Wuhan, China; 2 Department of Gastrointestinal Surgery, The Sixth Hospital of Wuhan, Affiliated Hospital of Jianghan University, Wuhan, China

**Keywords:** Hypertrophic cardiomyopathy, Heart failure, Diagnostic model, Nomogram

## Abstract

Heart failure (HF) manifests variably in patients with hypertrophic cardiomyopathy (HCM). This study developed and validated a diagnostic nomogram that uses routine clinical indicators to identify HF in individuals with HCM. Independent diagnostic indicators of HF in patients with HCM included B-type natriuretic peptide (BNP) (OR = 1.003, *P* < 0.001), diuretics (OR = 3.69, *P* = 0.014), and myocardial ischemia (OR = 4.99, *P* = 0.002). The diagnostic model demonstrated robust performance in both the training set and the validation set, yielding AUCs of 0.917 (95% CI: 0.879–0.955) and 0.929 (95% CI: 0.873–0.984), respectively. At an optimal threshold of 0.157, the training set exhibited sensitivity and specificity of 89.1% and 79.9%, respectively, while the validation set showed sensitivity and specificity of 90.9% and 82.9%, respectively. The calibration curve indicated a good fit, and DCA revealed that the model provided a net clinical benefit. In this study, the developed nomogram model accurately identifies HF in patients with HCM, aiding in diagnostic stratification and personalized management.

*Clinical Trial Registration* This study was registered retrospectively at the Chinese Clinical Trial Registry (http://www.chictr.org.cn/), registration number ChiCTR2500106648 (Registration Date: 2025-07-28).

## Introduction

Hypertrophic Cardiomyopathy (HCM) is an autosomal dominant inherited cardiomyopathy primarily caused by mutations in sarcomere coding genes, characterized by asymmetric left ventricular hypertrophy. Its global prevalence ranges from 1:200 to 1:500 [1], and is a leading cause of sudden cardiac death in adolescents and athletes [2]. Despite advancements in diagnosis and treatment, the long-term prognosis of HCM remains challenged by various complications, with heart failure (HF) being the most common and impactful on patients’ quality of life and survival [3, 4]. HF represents a critical clinical outcome of HCM progression, presenting as HF with reduced or preserved ejection fraction, significantly elevating readmission rates and mortality risks [5, 6]. Currently, the assessment of HF risk in HCM patients relies mainly on empirical judgment or individual clinical and imaging parameters, such as left ventricular ejection fraction or New York Heart Function Classification, lacking systematic and quantitative multifactorial diagnostic model tools in clinical practice. While previous research has investigated the correlation between biomarkers or imaging parameters like high-sensitivity troponin and delayed gadolinium enhancement of cardiac magnetic resonance with prognosis [7–9], these markers are often expensive, not widely adopted, and have not been integrated into a clinical risk assessment model. Furthermore, the risk assessment tools commonly utilized in current international guidelines primarily concentrate on evaluating the risk of sudden cardiac death and guiding the implantation of implantable cardioverter-defibrillators [10, 11], failing to offer independent and precise predictions for HF, a prevalent and significant outcome. The absence of such predictive instruments poses substantial challenges in identifying high-risk patients early on and implementing risk-tailored intensive monitoring and interventions, consequently impeding the progress of personalized treatment and care.

This study aims to construct and validate a diagnostic nomogram that utilizes routine clinical, laboratory, and echocardiographic parameters to identify HF in patients with HCM. LASSO regression and multivariate logistic regression methods were employed to identify key diagnostic indicators, while a visual nomogram and interactive dynamic tools were developed to enhance the model’ s clinical applicability. The primary objective of this study is to provide a reliable and intuitive quantitative tool for the timely diagnosis of HF in HCM patients, thereby facilitating appropriate management.

This nomogram fills a critical gap in the management of hypertrophic cardiomyopathy (HCM) by offering a quantitative tool specifically for assessing heart failure (HF) risk, an area where existing sudden cardiac death (SCD) risk scores are inadequate. Its introduction provides substantial added value, as no guideline-recommended quantitative tools for HF risk assessment currently exist.

## Materials and methods

### Determine the study population and inclusion criteria

This single-center retrospective case–control study aimed to develop and validate diagnostic nomograms to identify HF in patients with HCM. All clinical, laboratory, and echocardiographic variables were collected at admission. HF status was determined from the same admission assessment, including clinical symptoms, signs, and echocardiographic findings. The study included 311 HCM patients admitted to the Cardiology Department of Wuhan Central Hospital between December 2022 and December 2024. A total of 366 inpatients with HCM were screened. 55 cases were excluded (including 39 cases due to incomplete clinical data and 16 cases due to combined diseases in the exclusion criteria), and finally 311 cases were included. No follow-up was conducted because this was a diagnostic study. HCM diagnosis adhered strictly to the 2023 ESC guidelines for cardiomyopathy management [12]. The study protocol received approval from the Medical Ethics Committee of Wuhan Central Hospital (approval number: WHZXKYL-2025-043) on March 15, 2025. Because this was a retrospective study using anonymized medical records, ethical approval was obtained after data collection had been completed. The study complied with the ethical standards of the Helsinki Declaration (revised in 2013) [13]. Inclusion criteria comprised age > 18 years; meeting HCM diagnostic criteria (confirmed by echocardiography or cardiac magnetic resonance imaging, with unexplained left ventricular wall thickness in any segment ≥ 15 mm, or left ventricular wall thickness ≥ 13 mm in confirmed pathogenic gene mutation carriers or their first-degree relatives); and absence of HF (baseline left ventricular ejection fraction ≥ 50% and no HF clinical symptoms/signs). HF diagnosis was based on the 2023 ESC guidelines for the management of cardiomyopathies, which includes patients with preserved ejection fraction (HFpEF, LVEF ≥ 50%) as well as those with reduced ejection fraction (HFrEF, LVEF < 50%). HF was diagnosed by two independent cardiologists during hospitalization using the 2023 ESC criteria, and the differences were resolved by a third evaluator. Exclude outpatient diagnosis. Exclusion criteria comprised incomplete clinical medical records, active malignant tumors with expected survival < 1 year, congenital heart disease, primary severe valvular disease (e.g., rheumatic mitral stenosis, severe aortic stenosis), active infection, acute myocardial infarction, myocarditis, pericarditis, and other clear causes of acute HF.

### Sample size calculation

This case-control study focused on HF in HCM patients as the primary outcome, guided by prior literature and our hospital’s preliminary data. Using a significance level of α = 0.05 (two-tailed) and test power of 1-β = 0.90, based on the preliminary data of our hospital, it is estimated that the proportion of HF cases among all HCM patients is 30%, with an allowable error of 6%. The calculated minimum sample size was 239 cases. To ensure model internal validation, approximately 30% additional cases were included, resulting in a total of 311 patients.

### Data collection

The clinical data of all eligible patients were gathered using standardized case report forms, encompassing demographic information (gender, age), clinical characteristics, and medical history (e.g., hypertension, diabetes, coronary artery disease, atrial fibrillation, syncope history), laboratory test results (BNP, troponin I, red blood cell distribution width standard deviation, lactate dehydrogenase) obtained within 24 h of admission, medication regimens (administration of β-blockers and diuretics), imaging findings and cardiac function parameters (e.g., left atrial diameter, left ventricular diameter, interventricular septal thickness, left ventricular posterior wall thickness, left ventricular ejection fraction, left ventricular outflow tract obstruction, apical hypertrophy), as well as electrocardiogram findings (atrial fibrillation and myocardial ischemia). Myocardial ischemia is defined as ST-segment depression ≥ 0.1 mV or T-wave inversion in at least two adjacent leads on a standard 12-lead electrocardiogram, excluding patients with coronary artery stenosis ≥ 50% documented by prior angiography.

### Statistical analysis

All analyses were conducted using R software (version 4.4.2). A significance level of *P* < 0.05 was deemed statistically significant. Measurement data following a normal distribution were presented as mean ± standard deviation, and group comparisons were performed using the independent sample t-test. Non-normally distributed data were presented as median (interquartile range), and group comparisons were conducted using the Mann-Whitney U test. Count data were expressed as the number of cases (percentage), and group comparisons were carried out using either the chi-square test or Fisher’s exact probability method.

### Predictor variable screening and model construction

To prevent overfitting of high-dimensional data, we employed least absolute shrinkage and selection operator regression (LASSO) to screen all candidate variables using the glmnet package [14]. The optimal penalty coefficient λ was determined through 10-fold cross-validation. The variable set corresponding to ‘lambda.1se’ was chosen to enhance the model’s generalization ability. Variables selected by LASSO were integrated into univariate logistic regression. Variables with *P* < 0.1 were retained in the multivariate logistic regression model. Final independent diagnostic indicators were selected using forward-backward stepwise regression based on the AIC criterion. Variables with *P* < 0.05 were retained in the final nomogram. Variables with 0.05 ≤ *P* < 0.1 are reported for transparency in table but were not included in the nomogram. Variables with *P* ≥ 0.1 were excluded. Subsequently, a nomogram was developed based on the results of multivariate logistic regression using the rms package. An interactive dynamic nomogram was created with the DynNom package to improve the clinical application’s convenience and practicality.

### Performance evaluation and verification of the model

The samples were randomly divided into training and validation sets in a 7:3 ratio. The model’s discrimination, calibration, clinical practicability, and rationality were assessed individually. The discrimination was evaluated by plotting the receiver operating characteristic curve and calculating the area under the curve (AUC). AUC values between 0.70 and 0.80 indicated moderate discrimination, while AUC values exceeding 0.80 indicated higher discrimination [15]. The optimal cut-off value was determined using the Youden index (the sum of sensitivity and specificity minus 1), and sensitivity and specificity were computed. The calibration curve and the Hosmer-Lemeshow test were utilized to assess the consistency between predicted and actual probabilities. The Brier score and p-value (*P* > 0.05 indicating good calibration) were computed to quantitatively assess calibration. The decision curve was employed to analyze the net benefit of the model across various threshold probabilities. Internal validation was conducted using 500 iterations of bootstrap resampling to compare the recognition performance of the training and validation sets using the Delong test.

## Results

### Comparison of patient characteristics and baseline data

A total of 311 patients diagnosed with HCM were randomly assigned to either the training set (*n* = 219) and the validation set (*n* = 92). The total cohort consisted of 195 (62.7%) male and 116 (37.3%) female patients. Among the entire cohort, 77 HCM patients were diagnosed with HF based on the admission evaluation. A comparative analysis of the baseline characteristics between the HF and non-HF groups in both the training and validation sets is presented in Table 1. Significantly higher ages were observed in the HF group compared to the non-HF group in both the training and validation sets (both *P* < 0.05). Regarding medical history, the prevalence of atrial fibrillation (AF) in HCM patients with HF was notably higher (training set: 50.9% vs. 14.6%; validation set: 50.0% vs. 10.0%, both *P* < 0.001), along with a significant increase in diuretic usage (both *P* < 0.001). In the validation set, the prevalence of diabetes was lower in the HF group than in the non-HF group (9.09% vs. 35.7%, *P* = 0.034), which contrasts with the pattern observed in the training set and with published literature. This discrepancy likely reflects sampling variability arising from the small size of the validation cohort (HF = 22 cases). Laboratory findings revealed a significant elevation in plasma B-type natriuretic peptide (BNP) levels among HF patients in both groups (all *P* < 0.001).Simultaneously, the HF group exhibited significant decreases in hemoglobin and platelet counts, alongside a notable increase in the standard deviation of red blood cell distribution width (RDW-SD) (all *P* < 0.01 in the training set), indicating the presence of anemia and inflammation. In the training set, the HF group exhibited elevated levels of lactate dehydrogenase (LDH) and α-hydroxybutyrate dehydrogenase (α-HBDH) compared to the non-HF group (*P* < 0.01). Additionally, α-HBDH levels were also higher in the validation set (*P* = 0.008). Conversely, troponin I did not show a significant difference between the groups in the training set (*P* = 0.129). The ratio LDH/α-HBDH was deemed a non-specific marker. Therefore, caution is advised in interpreting these results as indicative of myocardial injury. Furthermore, individuals in the HF group displayed lower triglyceride levels (training set *P* = 0.011, validation set *P* = 0.004), reduced estimated glomerular filtration rate (eGFR), and elevated blood urea nitrogen in the training set (all *P* < 0.05). Evaluation of cardiac ultrasound parameters revealed more pronounced right heart remodeling in HF patients, as evidenced by significantly increased right atrial diameter (RAD), right ventricular diameter (RVD), and main pulmonary artery (MPA) diameter in both the training and validation sets (all *P* < 0.05). Left ventricular ejection fraction (LVEF) was notably lower in the HF group of the training set (55.2% vs. 60.7%, *P* < 0.001). Concerning complications, the prevalence of HF combined with myocardial ischemia was higher in the training group (78.2% vs. 40.9%, *P* < 0.001), and the incidence of AF was significantly elevated in both groups (all *P* < 0.05).

**Table 1:** Baseline characteristics of patients with HCM

Variable	Training set			Validation set		
	Non-HF (*n* = 164)	HF (*n* = 55)	*p*.overall	Non-HF (*n* = 70)	HF (*n* = 22)	*p*.overall
Sex, (n, %)			0.118			0.225
Female	59 (36.0%)	27 (49.1%)		20 (28.6%)	10 (45.5%)	
Male	105 (64.0%)	28 (50.9%)		50 (71.4%)	12 (54.5%)	
Age, years, (n, %)	65.0 [57.0;72.0]	74.0 [67.0;84.0]	< 0.001	66.0 [61.0;73.0]	72.5 [68.2;81.0]	0.020
Hypertension, (n, %)	119 (72.6%)	40 (72.7%)	1.000	43 (61.4%)	18 (81.8%)	0.132
Diabetes, (n, %)	47 (28.7%)	20 (36.4%)	0.366	25 (35.7%)	2 (9.09%)	0.034
History of Coronary Heart Disease, (n, %)	75 (45.7%)	33 (60.0%)	0.094	32 (45.7%)	10 (45.5%)	1.000
History of Smoking, (n, %)	47 (28.7%)	17 (30.9%)	0.884	25 (35.7%)	7 (31.8%)	0.938
History of Alcoholism, (n, %)	25 (15.2%)	13 (23.6%)	0.224	7 (10.0%)	2 (9.09%)	1.000
History of Syncope, (n, %)	8 (4.88%)	3 (5.45%)	1.000	0 (0.00%)	3 (13.6%)	0.012
Family History of SCD, (n, %)	0 (0.00%)	0 (0.00%)	.	0 (0.00%)	1 (4.55%)	0.239
History of AF, (n, %)	24 (14.6%)	28 (50.9%)	< 0.001	7 (10.0%)	11 (50.0%)	< 0.001
β-blockers, (n, %)	77 (47.0%)	33 (60.0%)	0.129	31 (44.3%)	11 (50.0%)	0.823
Diuretics, (n, %)	14 (8.54%)	18 (32.7%)	< 0.001	4 (5.71%)	10 (45.5%)	< 0.001
Cardiac Pacemaker Implantation, (n, %)	5 (3.05%)	8 (14.5%)	0.004	3 (4.29%)	1 (4.55%)	1.000
BNP, pg/mL	113 [47.5;261]	633 [442;1116]	< 0.001	121 [74.4;229]	722 [553;1173]	< 0.001
Troponin I, ng/mL	0.03 [0.01;0.13]	0.04 [0.01;0.12]	0.129	0.03 [0.01;0.08]	0.04 [0.02;0.18]	0.075
LDH	179 [158;211]	206 [175;237]	0.002	183 [158;218]	208 [166;242]	0.092
α- HBDH, U/L	130 [115;150]	147 [122;178]	0.004	129 [111;148]	148 [128;178]	0.008
CK-MB, U/L	13.0 [9.25;16.0]	11.9 [9.25;14.7]	0.131	11.1 [9.90;15.0]	13.5 [10.2;16.8]	0.228
Creatine Kinase, U/L	94.9 [68.3;131]	68.0 [51.0;92.4]	< 0.001	77.4 [58.8;120]	81.7 [58.2;141]	0.749
Aspartate Aminotransferase, U/L	26.5 (16.6)	47.2 (150)	0.314	25.0 (11.0)	28.0 (14.6)	0.369
Creatinine, µmol/L	77.8 [62.6;94.9]	85.5 [72.2;107]	0.070	74.6 [65.7;90.2]	80.6 [66.7;112]	0.238
Uric Acid, µmol/L	379 (99.3)	396 (182)	0.531	390 (111)	396 (127)	0.847
Blood Urea Nitrogen, mmol/L	6.98 (3.32)	8.26 (4.20)	0.044	6.57 (2.43)	8.45 (4.45)	0.069
eGFR, mL/min/1.73 m²	81.6 (25.2)	70.2 (19.9)	0.001	84.6 (22.0)	76.0 (25.1)	0.158
Triglycerides, mmol/L	1.93 (1.56)	1.50 (0.86)	0.011	1.62 (0.95)	1.07 (0.69)	0.004
HDL, mmol/L	1.28 (0.95)	1.30 (0.62)	0.872	1.23 (0.45)	1.21 (0.56)	0.915
LDL, mmol/L	2.30 (0.77)	2.14 (0.75)	0.171	2.44 (0.87)	2.10 (0.90)	0.123
Apolipoprotein A1, g/L	1.24 [1.08;1.38]	1.26 [1.14;1.33]	0.839	1.25 [1.15;1.44]	1.21 [1.11;1.26]	0.052
Apolipoprotein B, g/L	1.07 (0.66)	0.99 (0.65)	0.385	1.13 (0.65)	0.93 (0.67)	0.230
Free Fatty Acids, mmol/L	0.83 (0.76)	0.73 (0.60)	0.326	0.80 (0.80)	0.62 (0.58)	0.273
Total Cholesterol, mmol/L	3.95 (1.09)	3.64 (0.88)	0.041	4.06 (1.02)	3.63 (1.22)	0.147
Non-HDL-C, mmol/L	2.80 [2.80;2.80]	2.80 [2.38;2.80]	0.122	2.80 [2.67;2.86]	2.80 [2.03;2.80]	0.150
hs-CRP, mg/L	7.71 (11.1)	10.2 (14.1)	0.240	8.92 (16.9)	9.68 (8.72)	0.782
Hemoglobin, g/L	136 (20.4)	127 (19.0)	0.003	140 (19.0)	127 (16.2)	0.003
Platelet Count, ×10⁹/L	203 (57.5)	169 (43.1)	< 0.001	203 (53.2)	168 (56.1)	0.014
Lymphocyte Count, ×10⁹/L	2.62 (12.7)	1.24 (0.66)	0.168	1.62 (0.66)	1.47 (0.77)	0.413
Neutrophil Count, ×10⁹/L	5.33 (6.25)	4.85 (2.28)	0.410	6.24 (9.59)	5.36 (2.99)	0.508
Monocyte Count, ×10⁹/L	0.76 (0.74)	0.65 (0.63)	0.288	0.87 (1.12)	0.76 (0.80)	0.599
Eosinophil Count, ×10⁹/L	0.51 (0.81)	0.32 (0.63)	0.081	0.51 (0.80)	0.40 (0.74)	0.573
Basophil Count, ×10⁹/L	0.46 (1.05)	0.29 (0.70)	0.195	0.42 (0.83)	0.32 (0.78)	0.640
RDW-SD, fL	43.8 (3.30)	47.6 (4.73)	< 0.001	44.3 (3.24)	46.7 (5.39)	0.060
White Blood Cell Count, ×10⁹/L	7.16 (2.78)	6.76 (2.53)	0.327	6.85 (2.71)	7.06 (3.11)	0.773
D-dimer, mg/L	0.96 (1.08)	1.03 (1.10)	0.686	0.82 (0.75)	1.20 (1.42)	0.236
Aortic Annulus Diameter, cm	3.42 (0.49)	3.38 (0.64)	0.692	3.27 (0.44)	3.59 (0.40)	0.003
Left Atrial Diameter, cm	4.07 (3.04)	4.32 (0.66)	0.332	3.91 (0.57)	4.50 (0.74)	0.002
Left Ventricular Diameter, cm	4.73 (3.63)	4.78 (0.86)	0.875	4.53 (0.63)	4.63 (0.38)	0.349
IVS, cm	1.52 (0.95)	1.59 (0.40)	0.438	1.53 (0.47)	1.55 (0.46)	0.912
LVPW, cm	1.20 (0.33)	1.19 (0.24)	0.836	1.26 (0.55)	1.16 (0.34)	0.282
MPA diameter, cm	2.30 [2.10;2.40	2.40 [2.25;2.65]	< 0.001	2.30 [2.10;2.40]	2.40 [2.30;2.58]	0.004
RAD, cm	3.55 [3.30;3.80]	3.80 [3.50;4.40]	< 0.001	3.50 [3.20;3.77]	3.80 [3.62;4.20]	< 0.001
RVD, cm	3.20 [2.90;3.50]	3.40 [3.10;3.70]	0.013	3.10 [2.90;3.40]	3.45 [3.10;3.77]	0.003
AV Vmax, cm/s	142 [120;157]	133 [115;148]	0.155	136 [127;149]	146 [110;147]	0.851
AV PG, mmHg	8.00 [6.00;10.0]	7.00 [5.00;9.30]	0.126	7.00 [6.00;9.30]	8.00 [5.00;9.30]	0.876
PV Vmax, cm/s	102 (25.5)	94.7 (22.2)	0.035	101 (21.0)	95.8 (24.0)	0.392
PV PG, mmHg	4.88 (7.26)	3.40 (2.33)	0.023	3.95 (2.15)	3.64 (2.65)	0.618
E peak diastolic pressure gradient	2.09 (5.36)	2.85 (2.30)	0.148	1.68 (3.41)	2.07 (2.19)	0.530
LVEF, %	60.7 (5.04)	55.2 (8.80)	< 0.001	60.0 (5.40)	58.2 (7.42)	0.282
Cardiac Magnetic Resonance, (n, %)	12 (7.32%)	2 (3.64%)	0.526	3 (4.29%)	1 (4.55%)	1.000
LVOTO, (n, %)	32 (19.5%)	6 (10.9%)	0.210	8 (11.4%)	4 (18.2%)	0.471
Apical Hypertrophy, (n, %)	17 (10.4%)	2 (3.64%)	0.169	2 (2.86%)	3 (13.6%)	0.087
Ventricular Arrhythmia, (n, %)	48 (29.3%)	19 (34.5%)	0.571	17 (24.3%)	10 (45.5%)	0.102
Myocardial Ischemia, (n, %)	67 (40.9%)	43 (78.2%)	< 0.001	35 (50.0%)	15 (68.2%)	0.212
AF, (n, %)	33 (20.1%)	29 (52.7%)	< 0.001	13 (18.6%)	10 (45.5%)	0.024
Mitral Regurgitation, (n, %)	41 (25.0%)	15 (27.3%)	0.876	17 (24.3%)	7 (31.8%)	0.672

*BNP* B-type Natriuretic Peptide, *CK-MB* Creatine Kinase-MB, *eGFR* Estimated Glomerular Filtration Rate, *HDL* High-Density Lipoprotein, *LDL* Low-Density Lipoprotein, *hs-CRP* high-sensitivity C-Reactive Protein, *IVS* Interventricular Septal Thickness, *LVPW* Left Ventricular Posterior Wall Thickness, *AV Vmax* Aortic Valve Maximum Velocity, *PV Vmax* Pulmonary Valve Maximum Velocity, *LVOTO* Left Ventricular Outflow Tract Obstruction, *α-HBDH* α-Hydroxybutyrate Dehydrogenase, *Non-HDL-C* Non-High-Density Lipoprotein Cholesterol, *RDW-SD* Red Cell Distribution Width-Standard Deviation, *AV PG* Aortic Valve Pressure Gradient, *PV PG* Pulmonary Valve Pressure Gradient. *MPA* main pulmonary artery measured at end-diastole

### Predictive variable screening

**Fig. 1:**
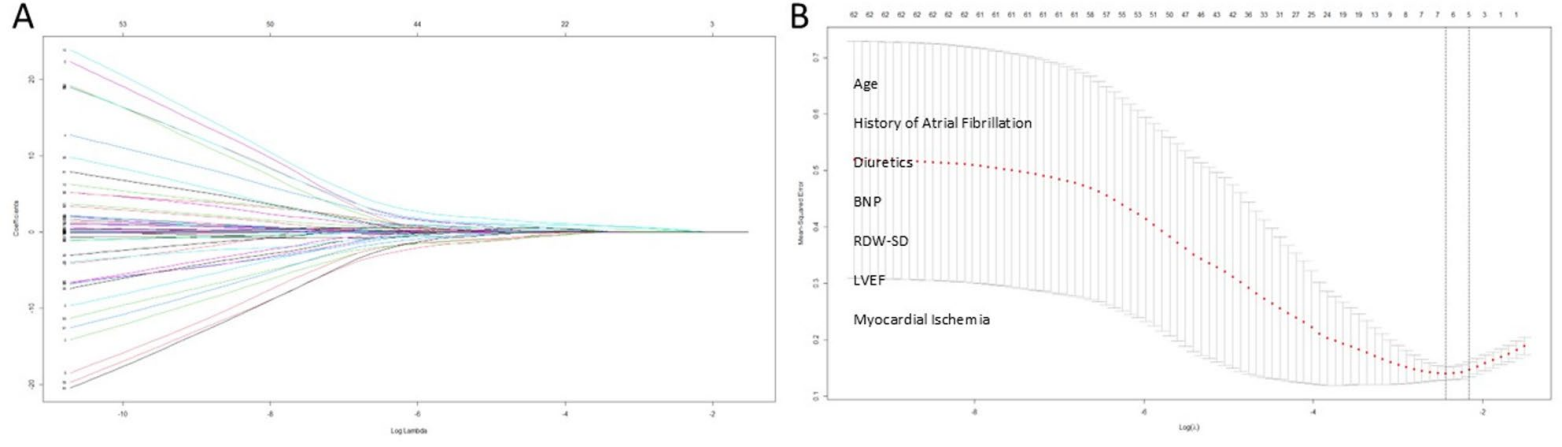
Illustrates the feature selection process utilizing the LASSO regression model within the features group. The LASSO coefficients resulting from the regression analysis are presented in **A**. Subsequently, a λ value of 0.09567 was selected through 10-fold cross-validation, as depicted in **B**, where the x-axis represents the optimal tuning parameter (Logλ) and the y-axis represents the binomial deviance. The dotted line indicates the positions of lambda.min and lambda.1se

To select the most predictive factors from numerous candidate variables and prevent model overfitting, we employed LASSO regression for initial feature screening. As the regularization parameter λ’s logarithmic value increases, the variable’s regression coefficient gradually diminishes towards zero (Fig. 1A). The optimal λ value (λ = 0.09567, Fig. 1B) was determined through 10-fold cross-validation, selecting the lambda.1se criterion to yield a more concise model. A total of 7 non-zero coefficient variables were identified as the core feature set for subsequent analysis, including Age, BNP, Diuretics, History of AF, LVEF, Myocardial Ischemia, and RDW-SD.

These 7 potential diagnostic indicators were then incorporated into the multivariate logistic regression model for analysis. The findings indicated that BNP level, diuretic use, and myocardial ischemia emerged as independent risk factors for HF in patients with HCM (*P* < 0.05). Variables with marginal or nonsignificant associations were excluded from the nomogram. Specifically, LVEF with *P* = 0.058 (*P* < 0.1) was not included. History of AF (*P* = 0.126), age, and RDW-SD with *P* ≥ 0.1 were also excluded. The specific parameters of each factor in both univariate and multivariate regression analyses can be found in Table 2. Ultimately, based on these three variables, they were identified as the fundamental indicators for developing the predictive model.

**Table 2:** Univariate and multivariable logistic regression analyses for HF in patients with HCM

Characteristics	Univariate logistic regression						Multivariate regression					
	B	SE	OR	CI	Z	*P*	B	SE	OR	CI	Z	*P*
Age	0.082	0.016	1.09	1.05–1.12	5.152	<0.001						
BNP	0.004	0.001	1.00	1-1.01	6.252	<0.001	0.003	0.001	1.003	1.002–1.005	4.492	<0.001
Diuretics	1.651	0.401	5.21	2.38–11.44	4.119	<0.001	1.305	0.534	3.69	1.3-10.51	2.445	0.014
History of AF	1.800	0.349	6.05	3.05–11.99	5.163	<0.001						
LVEF	-0.145	0.031	0.86	0.81–0.92	-4.638	<0.001	-0.074	0.039	0.93	0.86-1	-1.896	0.058
Myocardial Ischemia	1.646	0.363	5.19	2.55–10.57	4.534	<0.001	1.608	0.508	4.99	1.85–13.52	3.168	0.002
RDW-SD	0.259	0.049	1.3	1.18–1.43	5.281	<0.001						

### Model development and validation

**Fig. 2:**
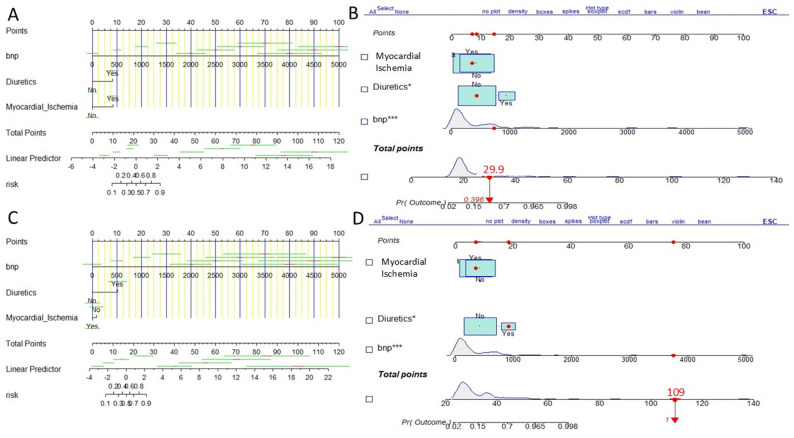
Displays the HF diagnostic nomogram. **A** and **C** represent the network nomogram in the training set and the validation set, respectively. **B** and **D** depict the interactive dynamic nomogram of the training set and the validation set, respectively

A nomogram model was developed based on three independent diagnostic indicators to estimate the risk of HF in patients with HCM (Fig. 2A). This model translates the regression coefficients from multivariate logistic regression into a user-friendly scoring system. Each predictor is assigned a score based on its contribution, and the cumulative score correlates directly with the individual probability of developing HF. To enhance the model’s clinical utility, an interactive dynamic nomogram was created (Fig. 2B). This tool enables real-time input of patient-specific parameters, facilitating instant calculation and visualization of their risk probability, thereby improving decision support convenience and flexibility. Internal validation data were used to construct a nomogram (Fig. 2C) and an interactive dynamic nomogram (Fig. 2D), further enhancing the model’s user-friendliness and widespread applicability.

### Performance evaluation and validation of the model

**Fig. 3:**
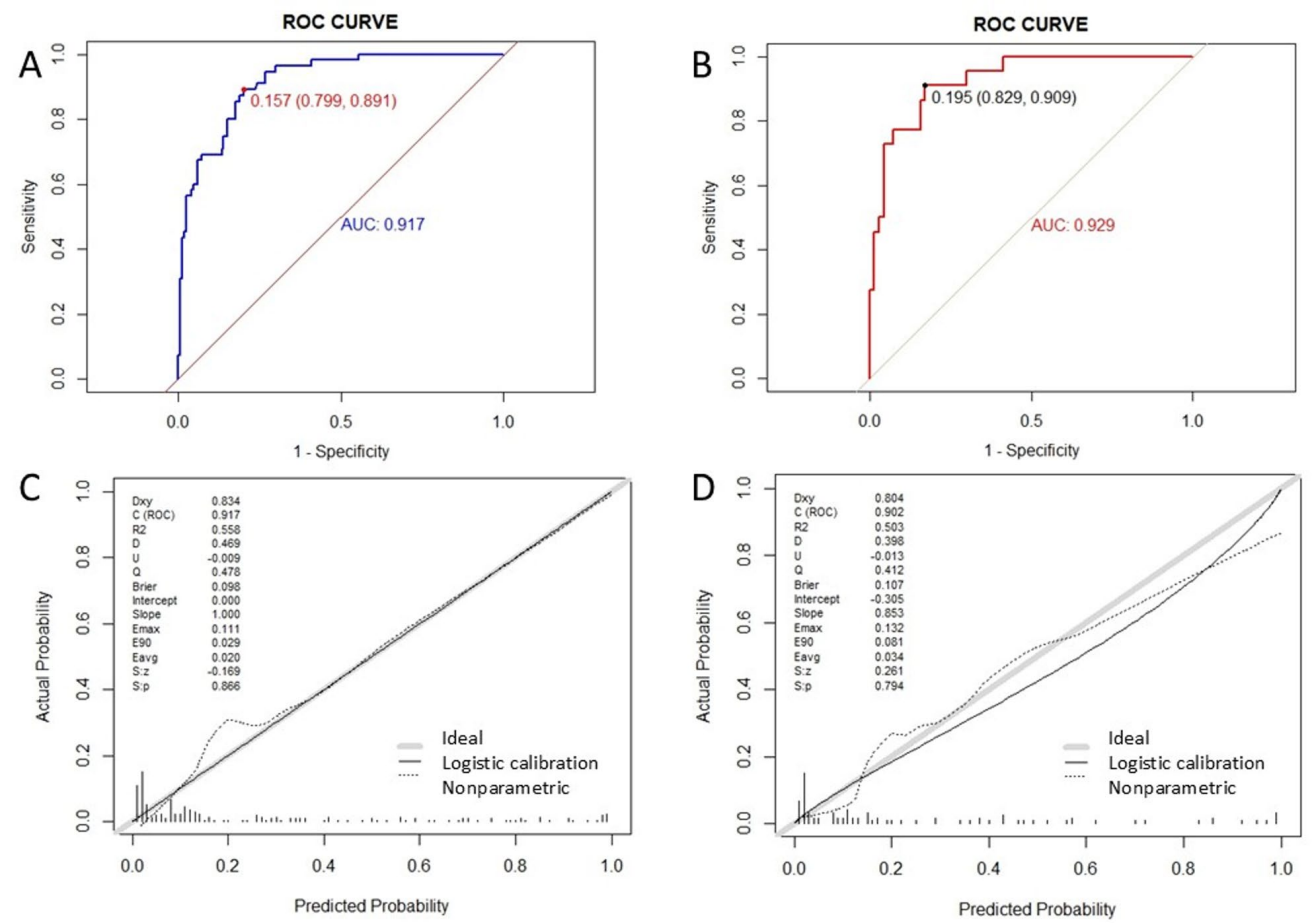
Illustrates the model’s discrimination and calibration. **A** and **B** depict the ROC curves for the training and validation sets, respectively. **C** and **D** show the calibration curves for the training and validation sets, respectively. The horizontal axis represents the predictive probability of HF in HCM, while the vertical axis represents the actual incidence of HF in HCM. The gray line signifies when the predictive probability equals the actual probability

**Fig. 4:**
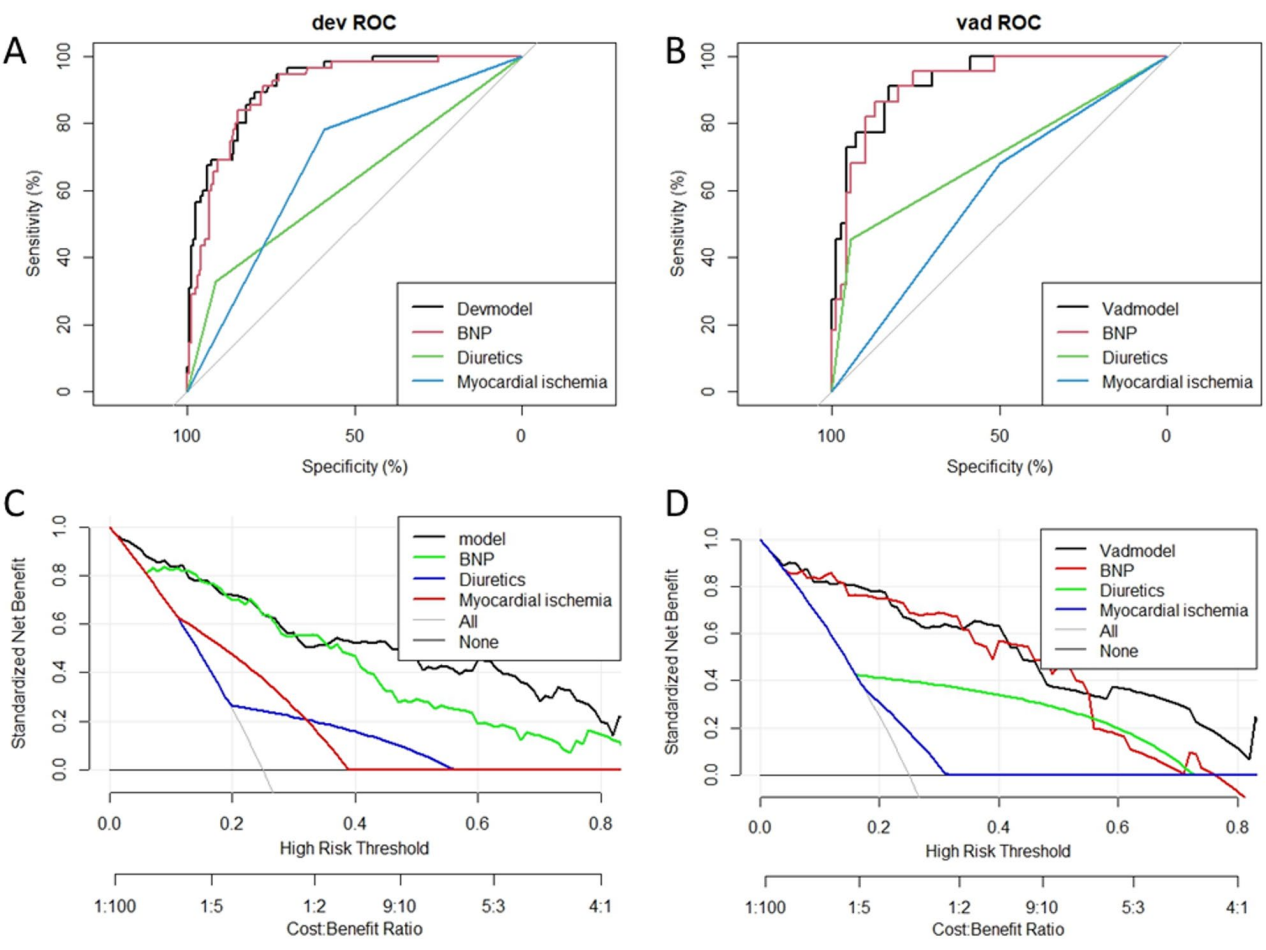
The model’s rationality is depicted in this figure **A**, **B** represent the ROC rationality curves for the training set and the validation set, respectively, while **C** and **D** represent the DCA rationality curves for the training set and the validation set, respectively

**Fig. 5:**
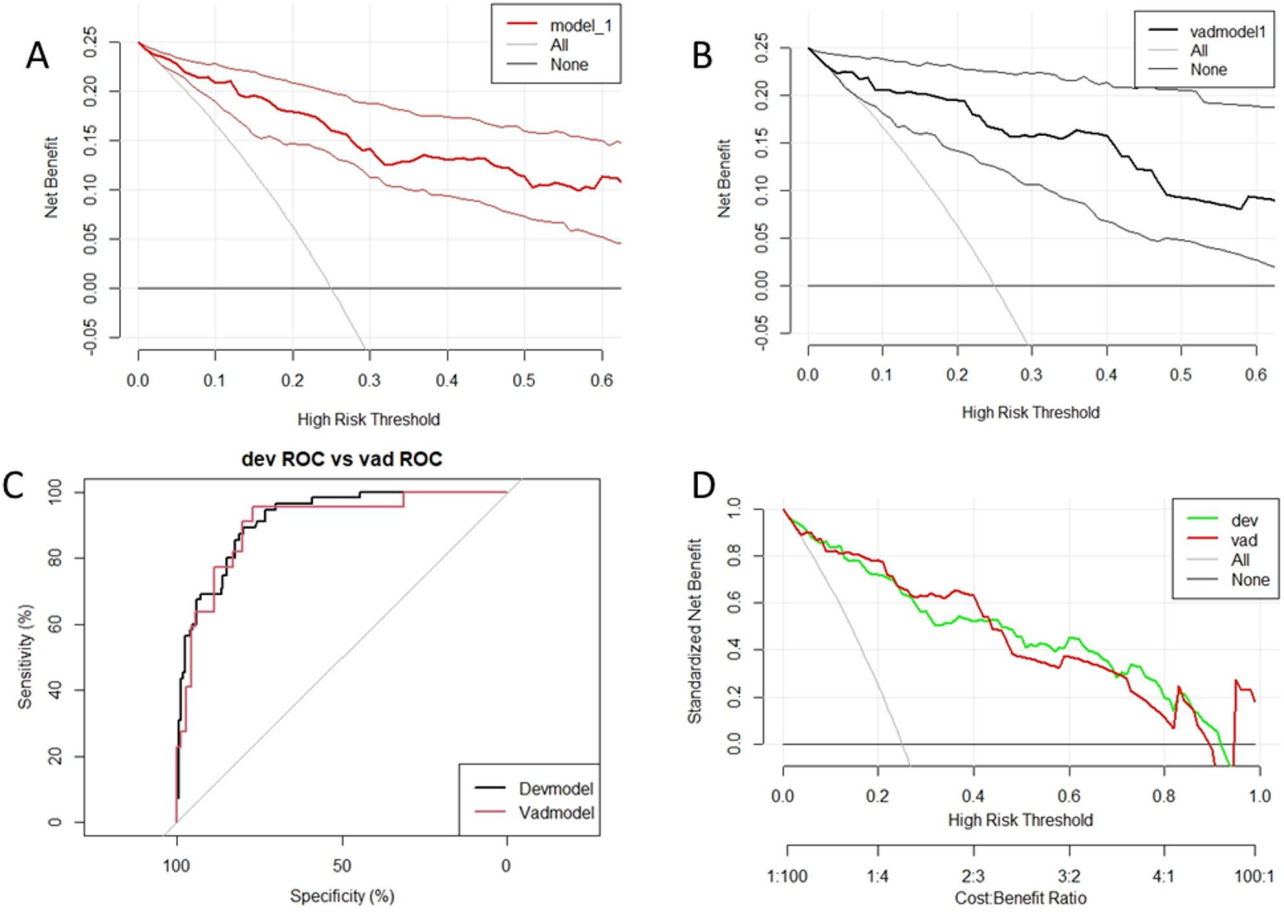
**A**, **B** The clinical applicability of the model, illustrating the DCA for the training and validation sets. The x-axis represents the threshold probability, while the y-axis displays the net benefit. The gray line signifies the scenario where all HF cases are positive, whereas the black line represents all negative HF cases. **C**, **D** The Delong test curves. The ROC curves in **C** for both the training and validation sets exhibit a high degree of overlap, indicating consistent model discrimination performance. In **D**, the decision curves for the training and validation sets demonstrate strong alignment, suggesting generalization of the model’s clinical net benefit

The model exhibits strong diagnostic discrimination in both the training and validation sets, with AUC values of 0.917 (95% CI: 0.879–0.955) and 0.929 (95% CI: 0.873–0.984) (Fig. 3A and B). At the optimal diagnostic threshold of 0.157, the training set demonstrated a sensitivity of 89.1% and a specificity of 79.9%, while the validation group showed a sensitivity of 90.9% and a specificity of 82.9%. The calibration curve indicates a high level of agreement between predicted and actual probabilities, closely aligning with the ideal diagonal (Fig. 3C and D). The Hosmer-Lemeshow test yielded non-significant results (all *P* > 0.05), and the Brier score was lower in the training set (0.098) compared to the validation set (0.107), further confirming the model’s excellent calibration. Decision curve analysis (DCA) revealed significant net benefits across a broad range of threshold probabilities for both the training set (2.5%-92%) and the validation set (5–90%) when utilizing this model for clinical decision-making (Fig. 5A and B). Rationality analysis (Fig. 4A–D) further confirmed the superiority of the comprehensive model that integrates BNP, diuretic use, and myocardial ischemia over individual indicators in terms of discriminant ability (ROC curve) and clinical net benefit (decision curve). This highlights the added diagnostic value of multi-factor integration. Bootstrap resampling (500 iterations) verified the stability of the model performance index. The Delong test indicated no significant difference in AUC between the training and validation sets (*P* > 0.05), with highly consistent standardized net benefit curves for both (Fig. 5C and D), demonstrating the model’s robustness and internal generalization ability.

## Discussions

This study has effectively developed and validated a diagnostic nomogram to identify HF in individuals with HCM using admission clinical data. By utilizing LASSO regression and multivariate logistic regression analyses, we have determined that BNP, Diuretics, and Myocardial Ischemia were independently associated with HF in HCM patients. The model exhibited outstanding discriminatory power, calibration, and clinical utility in both the training and validation datasets, offering a straightforward and efficient quantitative instrument for early risk assessment of HCM patients.

BNP emerged as a robust predictor of HF in this study (*P* < 0.001). BNP is primarily secreted by ventricular myocytes in response to increased wall stress, serving as a crucial marker for myocardial remodeling, diastolic dysfunction, and neuroendocrine system activation [16]. Our findings align closely with previous research indicating that elevated BNP levels are significantly associated with an increased risk of adverse cardiovascular events in patients with HCM [17–19]. This study revealed that BNP levels exceeding 633 pg/mL were significantly correlated with the incidence of HF, thereby providing a specific reference for clinical risk assessment [20]. The predictive value of using diuretics as a clinical intervention indicator (OR = 3.69) is significant, indicating volume overload or subclinical HF, a clinical sign of cardiac insufficiency [21]. The substantial contribution of diuretics in the model implies that their use may already signify potential hemodynamic disorders and HF risks, even without meeting the diagnostic criteria for overt HF. The study’s findings confirmed that myocardial ischemia is an independent risk factor for HCM-HF (OR = 4.99). In patients with HCM, myocardial ischemia primarily results from coronary microcirculation dysfunction rather than epicardial coronary artery stenosis [22]. This mechanism involves abnormal small artery structure in the myocardium, endothelial dysfunction, and reduced diastolic reserve, leading to significant impairment of coronary blood flow reserve [23, 24]. The chronic myocardial perfusion deficiency can induce myocardial cell damage, stimulate interstitial fibrosis, disrupt energy metabolism, and directly contribute to ventricular remodeling and deterioration of cardiac function [25].

This model integrates key indicators representing the three dimensions of neuroendocrine activation (BNP), volume load status (diuretic use), and myocardial oxygen supply-demand fundamental contradiction (myocardial ischemia). In contrast to assessment methods relying on a single indicator or costly complex tests (e.g., cardiac magnetic resonance), all variables in this model are readily obtainable in routine clinical practice and exhibit broad applicability. The model’ s outstanding performance (AUC > 0.90 in both the training and validation sets) implies that these three indicators collectively characterize the pathophysiological state preceding HF from a complementary perspective. Notably, myocardial ischemia, the most robust predictor, underscores the critical importance of early detection and intervention for HCM microcirculation dysfunction. The inclusion of diuretic use as an indicator introduces the innovative concept of utilizing clinical diagnostic and therapeutic actions as a risk cue, thereby enhancing the model’s practical utility.

In the discussion section, we have detailed the distinctions between our approach and existing SCD risk scores, emphasizing the innovative integration of three conventional indicators. Unlike the current HCM Risk stratification framework, which primarily focuses on SCD (e.g., HCM risk-SCD), our nomogram offers a novel, quantitative method for assessing HF. Although BNP and myocardial ischemia are individually associated with poor HCM prognosis, and diuretics provide clinical insights into volume overload, their combined use as diagnostic nomograms has not been previously documented. The strength of our model lies in its ability to integrate these three conventional indicators—two biomarkers/electrocardiogram manifestations and one clinical behavior indicator—into a single, user-friendly scoring system. This system can be easily applied at the bedside, eliminating the need for advanced imaging or genetic testing.

Although the model is diagnostic, it can inform early interventions to slow disease progression. For HCM patients whom the nomogram classifies as having a high probability of HF, clinicians should consider individualized physical therapy and supervised exercise prescriptions, avoid high-intensity exertion, and encourage safe aerobic activities to preserve functional capacity. The model also supports multidisciplinary discussions among cardiologists, rehabilitation specialists, and nurses to optimize pharmacotherapy (for example, use diuretics with caution and reduce afterload) and to schedule timely follow-up. Early identification of HF using this nomogram permits proactive management, which may decelerate disease progression and improve quality of life.

This study used a single-center retrospective design and performed rigorous internal validation (random split - sample, 500 times - iterative bootstration). However, it lacked an independent, prospective, multi-center external validation cohort. Therefore, the reported AUC values reflect only internal performance and may not generalize. In addition, all participants were of East Asian descent and recruited from Wuhan, China. Although the nomogram’ s clinical diagnostic indicators–BNP, diuretic use, and myocardial ischemia are widely available and biologically plausible markers of heart failure in HCM, their optimal diagnostic thresholds and model calibration may differ across populations with distinct genetic backgrounds, comorbidity profiles, and healthcare practices. Moreover, clinical thresholds for initiating diuretics vary between medical systems, which could limit the variable’ s transportability. Consequently, despite offering a practical, low-cost tool to identify HF in HCM patients, the model requires external validation in independent cohorts before clinical use, particularly in Egypt and other MENA populations. Prospective, multicenter external validation is needed to confirm generalizability and recalibrate thresholds if warranted. Other limitations are: reliance on complete-case analysis without imputation for missing data; absence of a predefined model-update strategy; and inability to perform NRI/IDI comparisons because no established HF risk tools exist for HCM. These issues should be addressed in future research.

This study employed a single-center retrospective design with rigorous internal validation; however, the sample’s representativeness may be limited. Future validation should involve multi-center, prospective cohorts to enhance the model’s generalizability. Furthermore, the model lacks parameters like delayed enhancement of cardiac magnetic resonance and genotypes, which could add value. Subsequent research could explore integrating these new markers with the model to create a more robust predictive system.

## Conclusion

This study established and validated a diagnostic nomogram based on traditional clinical variables. The model effectively identifies HF in HCM patients by incorporating three independent diagnostic indicators: BNP, diuretic use, and myocardial ischemia. It exhibits strong discriminative ability and clinical relevance, offering a practical tool for the timely diagnosis of HF in HCM patients and potentially optimizing clinical decision-making.

## Data Availability

Not applicable.
